# Amelioration of diet-induced nonalcoholic steatohepatitis in rats by Mn-salen complexes via reduction of oxidative stress

**DOI:** 10.1186/1423-0127-19-26

**Published:** 2012-02-29

**Authors:** Alireza Rezazadeh, Razieh Yazdanparast, Mahsa Molaei

**Affiliations:** 1 Institute of Biochemistry and Biophysics, University of Tehran, Tehran, Iran; 2 Research Center for Gastroenterology and Liver Disease, Shahid Beheshti University of Medical Sciences, Tehran, Iran

**Keywords:** Nonalcoholic fatty liver, superoxide dismutase mimetic, oxidative stress, Mn-salen complexes, methionine and choline deficient diet

## Abstract

**Background:**

Nonalcoholic steatohepatitis (NASH), a progressive stage of nonalcoholic fatty liver disease (NAFLD), is characterized by steatosis (accumulation of triacylglycerols within hepatocytes) along with inflammation and ballooning degeneration. It has been suggested that oxidative stress may play an important role in the progress of NAFLD to NASH. The aim of present study was to determine whether antioxidant supplementations using EUK-8, EUK-134 and vitamin C could improve the biochemical and histological abnormalities associated with diet-induced NASH in rats.

**Methods:**

NASH was induced in male N-Mary rats by feeding a methionine - choline deficient (MCD) diet. The rats were fed either normal chow or MCD diet for 10 weeks. After NASH development, the MCD-fed rats were randomly divided into four groups of six: the NASH group that received MCD diet, the EUK-8 group which was fed MCD diet plus EUK-8, the EUK-134 group which was fed MCD diet plus EUK-134 and the vitamin C group which received MCD diet plus vitamin C. EUK-8, EUK-134 and vitamin C (30 mg/kg body weight/day) were administered by gavage for eight weeks.

**Results:**

Treatment of MCD-fed rats with salens reduced the sera aminotransferases, cholesterol, low density lipoprotein contents, the extent of lipid peroxidation and protein carbonylation whereas the HDL-C cholesterol levels were significantly increased. In addition, EUK-8 and EUK-134 improved steatosis, ballooning degeneration and inflammation in liver of MCD-fed rats.

**Conclusion:**

Antioxidant (EUK-8, EUK-134 and vitamin C) supplementation reduces NASH-induced biochemical and histological abnormalities, pointing out that antioxidant strategy could be beneficial in treatment of NASH.

## Background

Nonalcoholic fatty liver disease (NAFLD) is regarded as a hepatic manifestation of the metabolic syndrome which is associated with obesity, insulin resistance, dyslipidemia and hypertension [1]. NAFLD encompasses a spectrum of liver abnormalities, including simple steatosis (nonalcoholic fatty liver, NAFL), nonalcoholic steatohepatitis (NASH), fibrosis and cirrhosis [2]. NAFLD is defined as hepatic fat accumulation exceeding 5-10% of liver weight, in the absence of excess alcohol consumption or any other liver disease and other causes of steatosis, such as certain toxins and drugs [2,3]. It is estimated that NAFLD affects 20-30% of the general population, 20-30% of which eventually progress into NASH. As a progressive stage of NAFLD, NASH is characterized by steatosis (accumulation of triacylglycerols within hepatocytes) along with inflammation and ballooning degeneration. The pathogenesis of steatosis occurrence and progression of NAFLD into NASH are not fully understood. A "multi-hit" (previously called " two-hit") hypothesis has been proposed to explain the progression of NAFLD into NASH [4]. It is believed that insulin resistance and/or prolonged over-nutrition leads to steatosis (first hit), which sensitizes the hepatocytes to other factors such as oxidative stress, cytokine/adipokine interplay and mitochondrial injury, eventually leading to the development of NASH [2,4]. Insulin resistance is caused by genetic and acquired factors (such as obesity, pregnancy, inactive lifestyle, etc.). Possibly, there is a link between oxidative stress and insulin resistance.

The incidence of oxidative stress has been reported to be high among patients with metabolic syndrome and NASH [5,6]. In these patients, superoxide dismutase activity, the serum vitamin C and α-tocopherol concentrations are low, whereas lipid peroxidation and protein carbonylation levels are high [7]. Lipid peroxidation products initiate chemical modification of many biological molecules and also they participate in signal transduction of many inflammatory responses with up-regulation of pro-inflammatory cytokines, such as TNF-α, IL-6 and IL-1. TNF-α in turn induces NADPH oxidase that leads to inflammation [8]. The involvement of oxidative stress in the pathogenesis of NASH, suggests that antioxidants might have beneficial effects in the treatment of NASH patients [7]. It has been shown that antioxidant drugs improve insulin resistance in the metabolic syndrome models. For instance, antioxidant vitamin (vitamins C and E) therapies of diabetic patients had improving effects on insulin resistance [8]. Antioxidant enzymes [e.g. superoxide dismutase (SOD) and catalase] protect cells against oxidative damage. However, the application of proteinaceous antioxidant enzymes might clinically be an inefficient approach due to enzyme instability and the high cost and thus, this approach has limited application [9]. On the other hand, the use of synthetic antioxidant enzyme mimetics might prove to be an ideal alternative approach for antioxidant therapy. To date, several types of SOD mimics have been evaluated using various experimental models of oxidative stress [10]. The Mn-salen complexes [salen = bis(salicylaldehyde)ethylenediamine], are amongst these mimetics. These low-molecular-weight synthetic compounds exhibit SOD, catalase and peroxidase activities [11]. The mechanism for the catalase activity of Mn-salen involves the oxidation of Mn(III) to oxomanganese by H_2_O_2_, releasing water. The oxomanganese is then reduced to Mn(III) by another hydrogen peroxide molecule to form oxygen and water. The mechanism by which each Mn-salen complex acts as a superoxide dismutase mimetic involves the reduction of Mn(III) to Mn(II) by O_2_^-· ^resulting in production of O_2_. The Mn(II) is oxidized back to Mn(III) by another molecule of O_2_^-· ^yielding H_2_O_2 _[12]. The protective effects of these antioxidants against oxidative stress-induced injuries have been reported in many *in vitro *and *in vivo *disease models, including, stroke, amyotrophic lateral sclerosis, Parkinson [13], excitotoxic neuronal injury [14] and adult respiratory distress syndrome [15].

**Figure 1:**
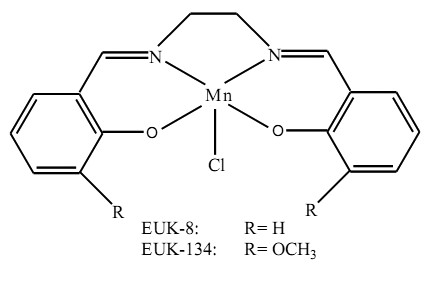
**Chemical structures of the Mn-salen complexes EUK-8 and EUK-134**.

Here, we report the effects of two Mn-salen complexes EUK-8 and EUK134 (Figure 1) on the treatment of NASH, induced by feeding rats a diet deficient in methionine and choline (MCD). We found that EUK-8 and EUK-134 treatments effectively normalized the indices of the oxidative stress, decreased the sera marker enzyme activities and also improved the liver morphology.

## Methods

### Materials

Bovine serum albumin (BSA), reduced nicotinamide adenine dinucleotide (NADH), reduced nicotinamide adenine dinucleotide phosphate (NADPH), ascorbic acid, 5, 5'-dithiobisnitro benzoic acid (DTNB), nitroblue tetrazolium (NBT), hydrogen peroxide (H_2_O_2_) and thiobarbituric acid (TBA) were obtained from Merck Co. (Darmstadt, Germany). Reduced and oxidized glutathione were obtained from Fluka (Buchs, Switzerland). Glutathione reductase (GR) was obtained from Sigma-Aldrich Chemical Co. Ltd. (Gillingham, England). Trichloroacetic acid (TCA), 2, 4-dinitrophenylhydrazine (DNPH) and Folin-Ciocalteu reagent were obtained from Sigma (St. Louis, MO, USA). All other chemicals were of analytical grade. Mn-salen compounds, EUK-8 and EUK-134, were synthesized as described by Baker *et al. *[16] and stored within a desiccator. Fresh solution of each Mn-salen was prepared in water as required.

### Animals, diets and drug treatments

Animal procedures were approved by the Ethics Committee of University of Tehran. Male N-marry rats (250-300 g) were obtained from Pasteur institute (Tehran, Iran). The rats were fed either normal chow or the MCD diet [17] for 10 weeks. After NASH development, the MCD-fed rats were randomly divided into four groups: the NASH group (n = 6) that received MCD diet, the EUK-8 group (n = 6) which was fed MCD diet plus EUK-8, the EUK-134 group (n = 6) which was fed MCD diet plus EUK-134 and the vitamin C group (n = 6) which received MCD diet plus vitamin C. The antioxidants, EUK-8, EUK-134 and vitamin C (30 mg/kg body weight/day) were administered orally for eight weeks. The Mn-salen derivatives were dissolved in double-distilled water and the NASH and the control groups received water as the vehicle. After eight weeks, the rats were sacrificed after an overnight fast, under diethyl ether anesthesia and the livers were rapidly removed and weighed. A portion of each liver was fixed in 10% formalin solution for histopathological examination and the remainder was snap-frozen for later analyses. All rats were weighed at the start of the experiment and before sacrifice to record the body weight variations.

### Assessment of steatohepatitis

Fresh liver tissue samples were kept in 10% formalin solution and paraffin blocks were subsequently prepared. Paraffin-embedded liver sections were stained with hematoxylin-eosin and Masson-trichrome. All specimens were evaluated blindly by an expert pathologist, using the scoring system proposed by Kleiner *et al. *[18]: steatosis (0-3), lobular inflammation (0-3) and ballooning degeneration (0-2). Fibrosis was minimal in all samples and was therefore not scored.

### Liver homogenate preparation

Each liver sample was homogenized in phosphate buffer (50 mM, pH 7.5) to obtain a 10% (w/v) liver homogenate. The homogenate was then centrifuged at 5000 ×*g *for 15 min at 4°C. Each supernatant was collected into 1 ml aliquots and stored at -70°C until use. The protein content of each sample was determined using Lowry method [19] and bovine serum albumin as the standard.

### Biochemical assays

The sera were separated by centrifugation at 4°C and stored at -20°C. The sera levels of total cholesterol (TC), high density lipoprotein cholesterol (HDL-C), triglycerides (TG), fasting blood glucose (FBG) and albumin were measured using commercially available kits (Pars Azmun, Tehran, Iran).

### Serum markers of hepatic damage

The sera activity of alkaline phosphatase (ALP), alanine aminotransferase (ALT), aspartate aminotransferase (AST) and gamma-glutamyl transferase (GGT), as some of the damage markers, were measured using commercially available kits (Pars Azmun, Tehran, Iran).

### Antioxidant enzyme assays

The liver superoxide dismutase activity was measured based on the inhibition of amino blue tetrazolium formazan formation in a reaction mixture composed of NADH, phenazine methosulfate (PMS) and nitroblue tetrazolium (NBT) [20]. The color intensity was measured using a spectrophotometer (VARIAN CARY 100 Conc, USA) at 560 nm. One unit of SOD is defined as the amount of protein that inhibits the rate of NBT reduction by 50%.

The catalase activity was measured by spectrophotometric analysis of the decomposition rate of hydrogen peroxide at 240 nm. The activity for H_2_O_2 _decomposition was expressed as *k*.s ^-1^mg^-1 ^protein, where *k *is the first-order rate constant [20].

### Hepatic markers of oxidative stress

Hepatic lipid peroxidation was determined using thiobarbituric acid reactive substances (TBARS) for the estimation of malondialdehyde (MDA) content. The absorbance was measured at 532 nm with respect to the blank solution. The concentration of TBARS was then calculated using an extinction coefficient of 155 (cm^-1 ^mM^-1^) for the TBARS/MDA complex [21].

Protein carbonyl (PCO) content was estimated using the 2, 4-dinitrophenylhydrazine (DNPH)-based procedure. The absorbance of each sample was measured at 320 nm and the tissue protein carbonyl content was calculated based on the molar extinction coefficient of DNPH (2.2 × 10^4 ^cm^-1 ^M^-1^) [22].

### Statistical analyses

All data were expressed as mean ± standard deviation (SD) and statistical significance of the differences between groups was assessed using Student's t-test. A level of *P *< .05 was regarded as statistically significant.

## Results

### Weight and liver index

The rats in NASH group showed a 12% decrease in body weight, but those in the normal diet group increased weight by 17%, whereas the body weights of the groups on EUK-8, EUK-134 and vitamin C increased by 10%, 15% and 8%, respectively. In contrast, the liver index [the percentage of wet liver weight to body weight (g)], reduced by EUK-8, EUK-134 and vitamin C treatments in MCD-fed group by 13%, 12% and 7%, respectively relative to drug - untreated MCD-fed rats (Table 1).

**Table 1:** Phenotypic and biochemical parameters.

	Control	NASH	MCD+EUK-8	MCD+EUK-134	MCD+Vit. C
**Body Weight (g)**					
Initial	265 ± 25	241 ± 20	251 ± 25	259 ± 21	242 ± 31
Final	310 ± 28	212 ± 21 *	276 ± 19 ^†^	298 ± 25 ^†^	261 ± 23 ^†^
**Liver index (%)**	3.01 ± 0.07	3.52 ± 0.09 *	3.09 ± 0.06 ^†^	3.11 ± 0.08 ^†^	3.27 ± 0.21 ^†^
**Serum**					
Albumin (g/dL)	3.27 ± 0.22	3.33 ± 0.16	3.17 ± 0.21	3.01 ± 0.11	3.41 ± 0.25
FBG (mM)	6.11 ± 0.18	11.91 ± 0.28 *	8.55 ± 0.18 ^†^	8.85 ± 0.21 ^†^	9.77 ± 0.23 ^†^
Total Cholesterol (mmol/L)	3.41 ± 0.19	5.55 ± 0.32 *	3.81 ± 0.14 ^†^	3.68 ± 0.18 ^†^	4.29 ± 0.24 ^†^
HDL-C (mmol/L)	2.21 ± 0.05	1.67 ± 0.06 *	2.81 ± 0.11 ^†^	3.04 ± 0.14 ^†^	2.36 ± 0.09 ^†^
LDL-C (mmol/L)	0.91 ± 0.06	1.49 ± 0.18 *	1.11 ± 0.08 ^†^	0.94 ± 0.06 ^†^	1.16 ± 0.07 ^†^
AST (U/L)	110.33 ± 5.23	177.12 ± 11.23 *	145.22 ± 8.33 ^†^	139.72 ± 12.98 ^†^	127.61 ± 14.53 ^†^
ALT (U/L)	67.32 ± 4.34	100.98 ± 9.22 *	86.17 ± 6.31 ^†^	80.11 ± 8.59 ^†^	76.07 ± 6.21 ^†^
ALP (U/L)	872.42 ± 71.25	1849.53 ± 89.21 *	1221.38 ± 65.29 ^†^	1134.15 ± 53.91 ^†^	1378.42 ± 72.78 ^†^
GGT (U/L)	9.61 ± 1.31	13.55 ± 1.18 *	10.85 ± 1.68 ^†^	10.09 ± 1.11 ^†^	10.37 ± 1.98 ^†^
**Liver**					
CAT (U)	2.65 ± 0.07	2.25 ± 0.09 *	2.78 ± 0.11 ^†^	3.23 ± 0.14 ^†^	2.91 ± 0.12 ^†^
SOD (U)	4.45 ± 0.16	3.38 ± 0.18 *	4.62 ± 0.21 ^†^	4.27 ± 0.16 ^†^	4.09 ± 0.21 ^†^
TBARS (nmol/mg liver protein)	0.34 ± 0.04	0.72 ± 0.05 *	0.52 ± 0.03 ^†^	0.48 ± 0.06 ^†^	0.45 ± 0.07 ^†^
PCO (nmol/mg liver protein)	1.41 ± 0.09	2.39 ± 0.11 *	1.94 ± 0.13 ^†^	2.07 ± 0.09 ^†^	1.81 ± 0.05 ^†^

Data are presented as means ± SD (n = 6) of triplicate measurements of each sample. * *P *< .05 compared to control. ^† ^*P *< .05 compared to NASH group.

### Histopathological analyses

**Figure 2:**
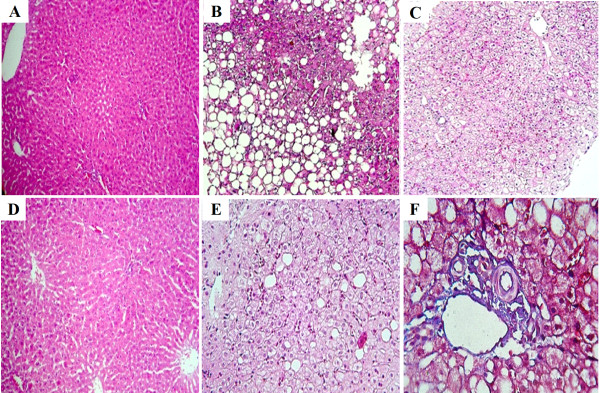
**Histological evaluation of rats' livers**. Photographs of tissues after Hematoxylin-eosin (A-E) or Masson trichrome staining (F). Original magnifications ×100. (A) Normal liver histology of a rat fed the standard diet. (B) MCD-fed diet (NASH group) showing ballooning degeneration, pericentral macrovesicular steatosis and lobular inflammation. (C) group MCD-fed diet + EUK-8 showing normal architecture with neither steatosis nor inflammation. (D) group MCD-fed diet + EUK-134 showing normal architecture with neither steatosis nor inflammation. (E) group MCD-fed diet + Vitamin C showing normal architecture, focal macrovesicular steatosis and no inflammation. No evidences of drug toxicity have been seen in all drug treated MCD-fed group. (F) No significant fibrosis is detected in MCD-fed group. MCD: methionine and choline deficient diet.

The histological studies of the liver samples from the control and the test groups were performed to investigate the occurrence of steatohepatitis. Treatment of MCD-fed rats with EUK-8, EUK-134 or vitamin C had significant effects on the grade of NASH among MCD-fed rats (Table 2). Based on Figure 2, the liver samples of the control animals showed no evidences of steatosis, inflammation or fibrosis (Figure 2A), the NASH group showed grade two liver steatosis, inflammation and ballooning degeneration. Furthermore, no incidence of fibrosis was recorded among the control and the MCD-fed rats (Figure 2, B and 2F). Normal liver architecture without the incidence of steatosis and inflammation was observed in EUK-8-treated rats (Figure 2C). Similar results were observed in EUK-134 group (Figure 2D). Among the vitamin C-treated rats however, focal macrovesicular steatosis without inflammation, were evident as shown in Figure 2E.

**Table 2:** Histopathological findings of liver tissue.

	Control	NASH	MCD+EUK-8	MCD+EUK-134	MCD+Vit.C
	(n = 5)	(n = 5)	(n = 5)	(n = 5)	(n = 5)
Steatosis (%)					
Grade 0	100	-	80	80	60
Grade 1	-	-	20	20	40
Grade 2	-	100	-	-	-
Ballooning (%)					
Grade 0	100	-	20	20	20
Grade 1	-	-	80	80	80
Grade 2	-	100	-	-	-
Inflammation (%)					
Grade 0	100	-	80	80	80
Grade 1	-	-	20	20	20
Grade 2	-	100	-	-	-
Fibrosis	-	-	-	-	-

All specimens were evaluated blindly by an expert pathologist, according to the scoring system proposed by Kleiner *et al. *[18]. MCD: methionie and choline deficient diet.

### Sera biochemical evaluations

Biochemical analyses of the sera were performed to document any possible changes in albumin, FBG and lipid profile of the control and the test animals. As summarized in Table 1, the albumin levels did not change among different groups of rats after eight weeks of the treatments. The sera levels of FBG, cholesterol and LDL-C significantly increased among the NASH group relative to the normal healthy rats by almost 95%, 63% and 63%, respectively, whereas the HDL-C cholesterol levels significantly decreased by 24% among the NASH group rats. The FBG levels were significantly reduced by EUK-8, EUK-134 and vitamin C treatments by about 55%, 50% and 35%. The cholesterol concentration was also reduced among the MCD rats under the effects of EUK-8, EUK-134 and vitamin C by 79%, 83%, and 65%, respectively. A significant elevation, however, was observed in the HDL-C level among the MCD rats treated with EUK-8, EUK-134 and vitamin C (by 51%, 62% and 31%, respectively), while the LDL-C concentration significantly decreased by 43%, 61% and 36%, respectively.

### Sera hepatic enzymes

In the NASH group, the activities of ALT, AST, ALP and GGT were significantly increased by 50%, 61%, 112% and 41%, respectively, relative to the control (Table 1). In the EUK-8 treated MCD rats, the activities of the aforementioned enzymes significantly reduced by 22%, 28%, 72% and 26%, respectively. Similar results were obtained for EUK-134 (31%, 33%, 82% and 34%, respectively). In the vitamin C-treated MCD rats, the activities of these enzymes were reduced by 36%, 44%, 53% and 32%, respectively.

### Hepatic markers of oxidative stress

First, we measured the effects of induced oxidative stress on the extent of hepatic lipid peroxidation and protein carbonylation, which were assessed in terms of TBARS and PCO contents. The results indicated that MCD diet increased TBARS and PCO levels by112% and 71%, respectively relative to the normal diet. Then, we measured the same factors among the antioxidant (EUK-8, EUK-134 and vitamin C) -treated rats. Our data indicated that the extents of lipid peroxidation decreased by 57%, 69%, 77% and that of protein carbonylation was suppressed by 32%, 23%, 42%, respectively (Table 1).

### Hepatic antioxidant enzymes

As indicated in Table 1, the hepatic catalase (CAT) and SOD activities were decreased by 15% and 26%, respectively among rats with NASH compared to normal healthy rats. On the other hand, the liver CAT and SOD activities were augmented among the EUK-8, EUK-134 and vitamin C - treated rats, relative to the NASH group, by 20%, 37%, 25% and 28%, 20%, 16%, respectively.

## Discussion

To date, no effective therapy has been proposed for patients with NASH, the progressive stage of NAFLD [23]. The prevalence of NASH is estimated to be around 3.5-5% amongst Americans [23] and about 3.3% for Iranians [24]. It has been proposed that over-production of mitochondrial and cytoplasmic superoxide anion, the precursor of reactive oxygen species (ROS), has a pivotal role in NASH development [25]. Oxidative stress increases lipid peroxidation and activates kupffer cells leading to production of inflammatory cytokines, such as TNF-α and IL-6 [25]. Therefore, antioxidant therapeutic approaches might be beneficial to NASH patients. In this context, we assessed the relative effects of two Mn-salen complexes (in comparison to vitamin C) on NASH in MCD-fed rats. In the current study, steatohepatitis was induced by a MCD diet. MCD diet causes weight loss, oxidative stress, lipid peroxidation and protein oxidation [25]. Our findings demonstrated, for the first time, that EUK-8 and EUK-134 supplementation reduced NASH-induced abnormalities without adverse effects on rat's liver. It has been indicated that MCD diet suppressed hepatic SCD-1 (stearoyl-coenzyme A desaturase-1) gene, augmented fatty acid catabolism, increased energy expenditure, decreased adipose tissue mass, caused liver injury and induced a catabolic state by hepatic inflammation which caused loss of body weight [26,27]. In agreement with previous studies, our results showed that the MCD feeding led to weight loss. However, the application of this study to obesity-related NASH is limited by the fact that the metabolic derangements induced by MCD diet might only represent a unique form of lipodystrophy, with significant weight loss in combination with hepatic steatosis [26]. In addition, it has been shown that antioxidants have anti-inflammatory effects [28]. Furthermore, in this study, the liver pathological assessments of MCD fed rats showed that MCD-induced inflammatory damage were attenuated in Mn-salen complexes. Apparently, the beneficial effect of Mn-salen complexes (as known catalytic antioxidants) on weight gain is related to their anti-inflammatory activity.

Our results revealed that MCD diet significantly enhanced the sera levels of AST, ALT, ALP and GGT, indicating considerable hepatocellular injury. Antioxidant (EUK-8, EUK-134 and/or vitamin C) treatments significantly reduced the sera activities of these enzymes, implying the hepatoprotective effects of EUK-8, EUK-134 and vitamin C against the harmful effects of MCD diet. These findings are in good agreement with Laurent *et al. *results [25] who showed that SOD mimics reduced the sera abnormalities in *ob*/*ob *mice. Furthermore, treatments with either EUK-8 or EUK-134 significantly lowered hepatic lipid peroxidation, PCO production and inflammation in MCD-fed rats, further confirming the anti-free radical activity of these compounds.

Administration of Mn-salen complexes to NASH rats not only suppressed the hepatic lipid peroxidation and protein oxidation, but also significantly increased the activity of antioxidant enzymes. It has been reported that superoxide anion and lipid peroxides inhibit catalase and SOD activities [29]. In the present study, we observed a decrease in the catalase and SOD activities in NASH rats compared to normal healthy rats. This observation is in agreement with the results published by Koruk *et al. *[30]. The products of lipid peroxidation have the ability to react with amino acid residues of antioxidant enzymes (catalase and SOD), leading to their inactivation. Suppression of lipid peroxidation by free-radical scavengers, such as antioxidants, might account for higher catalase and SOD activities among the drug - treated rats.

It has been demonstrated that NASH is associated with elevated levels of plasma cholesterol, triglycerides and LDL-C. In our study, the Mn-salen complexes exhibited significant hypolipidemic and hypocholesterolemic activities. The elevated level of LDL-C significantly reduced among rats with NASH following treatments with EUK-8 and EUK-134. This might be due to the antioxidant property of Mn-salen complexes and also due to the increased level of HDL-C, which is capable of inhibiting the LDL-C peroxidation and retarding the LDL-C accumulation. Remarkably, serum HDL-C level increased more than 50% both in the EUK-8 and EUK-134 treated NASH rats. HDL-C as a multifunctional lipoprotein, possess antioxidant and anti-inflammatory activities. The HDL-C antioxidant activity depends on different factors such as its chemical composition and the presence of associated enzymes such as paraoxonase 1 (PON1). The hydrolytic inactivation of LDL-C-oxidizing lipid peroxides by PON1 makes it a key player in the cellular defense against oxidative damage [31]. HDL-C, as an anti-inflammatory agent, restricts expression of pro-inflammatory cytokines such as tumor necrosis factor-α (TNF-α) and inhibits activation of nuclear factor kappa B (NF-kB) by interrupting a sphingosine kinase signaling pathway [32,33]. In addition, expression of the transforming growth factor (TGF)-β2, a cytokine with anti-inflammatory properties, is regulated by HDL-C [33]. Our results showed that MCD diet has a hyperglycemic effect which is reduced by administration of the Mn-salen complexes. It has been previously demonstrated that hyperglycemia promotes inflammatory responses through increasing mitochondrial superoxide production (activation of NF-kB and PKC) [34]. Considering these assumptions, the observed anti-inflammatory effects of the Mn-salen complexes might be due to their hypolipidemic and hypoglycemic properties.

## Conclusion

In conclusion, our findings clearly showed that EUK-8 and EUK-134 have hepatoprotective, hypolipidemic, hypocholesterolimic and hypoglycemic effects on MCD-fed rats. These activities were comparable to that of vitamin C, as a natural antioxidant. Thus, the results of this investigation might provide an insight into further application of Mn-salen complexes for human clinical studies for treatment of NASH. However, further investigations, regarding dose, bioavailability, metabolism, *in vivo *accumulation and the relevant cytotoxicity are required before moving toward clinical phase of investigation.

## Competing interests

The authors declare that they have no competing interests.

## Authors' contributions

All authors have made substantial contributions to the following: the conception and design of the study, analysis, interpretation of data, drafting the article and final approval of the version to be submitted.
